# A New Tool for Assessment of Professional Skills of Occupational Therapy Students

**DOI:** 10.3390/healthcare9101243

**Published:** 2021-09-22

**Authors:** Dulce Romero-Ayuso, Araceli Ortiz-Rubio, Paz Moreno-Ramírez, Lydia Martín-Martín, José Matías Triviño-Juárez, María Serrano-Guzmán, Enrique Cano-Detell, Erika Novoa-Casasola, Miguel Gea, Patrocinio Ariza-Vega

**Affiliations:** 1 Department of Physical Therapy, Occupational Therapy, Faculty of Health Sciences, University of Granada, 18016 Granada, Spain; aortiz@ugr.es (A.O.-R.); pazmor@ugr.es (P.M.-R.); lydia@ugr.es (L.M.-M.); msguzman@ugr.es (M.S.-G.); quicano@ugr.es (E.C.-D.); novoaerika@ugr.es (E.N.-C.); 2 Mind, Brain and Behavior Research Center (CIMCYC), University of Granada, 18011 Granada, Spain; 3 Primary Care Center Zaidín Center-East, Andalusian Health Service, 18006 Granada, Spain; jmtjuarez@hotmail.com; 4 Department of Preventive Medicine and Public Health, Faculty of Medicine, University of Granada, 18016 Granada, Spain; 5 Department of Computer Systems, Computer Sciences, University of Granada, 18014 Granada, Spain; mgea@ugr.es

**Keywords:** clinical reasoning, fieldwork, placement education, occupational therapy, communication skills, assessment, education

## Abstract

The assessment of the acquisition of professional skills is an essential process in occupational therapy students. Until now, there has been no standardized and validated instrument for evaluating these skills in Spanish occupational therapy students. This study reports the development and testing of the psychometric properties of the professional skills in students of occupational therapy during their practical training. Methods: A new instrument was developed to assess the professional skills of occupational therapy students, called CPTO. A total of 69 occupational therapists participated in evaluating 295 occupational therapy students from the University of Granada, between the 2018 and 2021 academic years. Results: Of a total of 79 items, the factor analysis yielded a final solution of 33 items, which explains 70.22% of the variance with the following three dimensions: (1) self-appraisal and professional responsibility (α = 0.951); (2) communication skills and delivering intervention (α = 0.944); and (3) clinical reasoning for assessing and planning the intervention (α = 0.947). The instrument allows students with low, medium, high and excellent clinical skills to be differentiated according to the cutting points established by the quartiles. Conclusion: the instrument has good psychometric properties, and is a useful tool to assess professional competencies in occupational therapy students during their practice placement education.

## 1. Introduction

Clinical practical training is an essential requirement in the curriculum of health science professionals [1]. It allows all the knowledge acquired during the theoretical training to be integrated and professional skills to be demonstrated in different clinical settings. According to the standards of the World Federation of Occupational Therapists, occupational therapy students are required to undertake a minimum of 1000 h of clinical practice, supervised by occupational therapists in different clinical and community settings [2].

During the period of practical training, learning and reasoning development as an occupational therapist becomes particularly relevant; in fact, 49% of clinicians identify clinical reasoning as the most important skill on which the training of occupational therapy students should focus [3].

Clinical reasoning (CR) has been defined as the practical ability that allows a general theory to be applied to a specific patient, in order to facilitate the highest level of functional independence to participate in daily life [3], and it is necessary for effective interventions [4,5]. It can also be understood as a process of solving cognitive problems about evaluation, interpretation of tests, treatment planning, treatment implementation, and evaluation of results. The learning of CR initially requires the following four steps: (1) acquisition of keys; (2) hypothesis generation; (3) interpretation of clues; and (4) hypothesis evaluation. For more experienced therapists, clinical reasoning often occurs as a process of pattern recognition [6] and “know-IN-action” [7].

All teaching–learning processes require an evaluation system to be established for competencies or skills. Therefore, it is essential to have evaluation tools that allow the development of professional skills in occupational therapy students to be known [8].

There are previous experiences in international universities, such as the University of Western Ontario, Canada. They developed an instrument named “competency-based fieldwork evaluation” (CBFE), which assesses students in different clinical settings, taking into account the following seven skills: (1) practice knowledge; (2) clinical reasoning; (3) facilitate change; (4) professional interactions; (5) communication; (6) professional development; and (7) performance management [8]. The CBFE was based on the previous instrument called occupational therapy student performance assessment (PEOTS), which included the following: (1) general communication; (2) professional relationships; (3) professional competence; (4) identification and evaluation of functions and needs; (5) program planning and development; (6) use of a therapeutic procedure; (7) select/use equipment and supplies, and select/use equipment and materials [8].

Other notable experiences are those carried out by the University of Queensland, who developed an instrument called the “modified fieldwork performance report” (MFPR), which was a revision of the fieldwork performance report (FPR), originally developed by the American Occupational Therapy Association [9]. The MFPR consisted of 59 items, which assessed the performance of the students at the halfway point and at the end of each internship [9]. The experience with this instrument showed that it was only adequate in the case of students who were carrying out practices in the hospital setting, with acute patients [9]. Likewise, the authors pointed out that the MFPR did not contemplate the evaluation of clinical reasoning, so the instrument was modified, in order to develop a more flexible and comprehensive instrument that would allow students in different contexts of occupational therapy practice to be evaluated. As a result of this modification, the student practice assessment form (SPEF) was created [9], which included the following eight dimensions: (1) professional practice; (2) self-management skills; (3) communication skills; (4) documentation; (5) assessment/information gathering; (6) intervention; (7) evaluation; and (8) group skills. In this instrument, it was contemplated that the learning goals could change in each environment. Therefore, they developed an item bank for each of the following practice settings: direct intervention, case management, and consultation or project development. In all cases, the objective of the SPEF was to be able to assess student learning and provide adequate feedback on their learning process. After SPEF, two reviews of this instrument have been performed, in 2011 [10] and in 2021 [11]. The last review included aspects related to cultural sensitivity, peer learning, informed consent, evidence-based practice, assistive technology, and environmental modifications, as well as the skills of students to manage their stress [11].

To the best of the authors’ knowledge, to date, there are no questionnaires available for the Spanish population that assess the acquisition of the professional competencies of occupational therapy students in practical training. In light of the above, and taking into account the relevance of assessing professional competencies during the fieldwork placement, we performed a study, which has the main aim to develop a new instrument to assess, in occupational therapy students, the professional competencies in their fieldwork, according to the legal and cultural framework in Spain, as well as to know its psychometric properties.

## 2. Materials and Methods

### 2.1. Design

A cross-sectional psychometric evaluation study was conducted. The study followed the methodology for the design and evaluation of the psychometric properties of a questionnaire [12,13].

### 2.2. Content Validity

For the development of this tool, a review of the literature was conducted by an expertise occupational therapist on assessment and another occupational therapist with 15 years of clinical practice experience, obtaining a pool of 79 items, with the following distribution: nine items in the “observation and evaluation” dimension, 13 items in the “planning intervention” dimension, 12 items in the “delivering intervention” dimension, six items in the “professional communication” dimension, 15 items in the “clinical responsibility” dimension, 14 items in the “self-appraisal and professional development” dimension and 10 items in the “evidence-based practice” dimension. After that, the coordinators of the practical training in occupational therapy, composed of three lecturers of this discipline (one was an expertise occupational therapist on assessment, the other was an occupational therapist with experience in the hospital setting and the third one was an occupational therapy lecturer specialist in theoretical frameworks), discussed these items and the format of the questionnaire. Subsequently, in the second week of September 2019, a first presential meeting with 58 occupational therapists (all of them were clinical practice instructors) in the Faculty of Health Sciences was organized by the coordinators of the practical training. The objective of this meeting was to explain the purpose of the study and review the questionnaire. Also, we aimed to know the opinion of the different clinical instructors about the items, length and format of the questionnaire. The entire questionnaire was jointly reviewed and any doubts that arose were answered. The agreed methodology for assessing the items followed was similar to other studies in health sciences [14,15]. Each item was reviewed, considering the following: (1) if competence was crucial for the successful performance of an occupational therapist; (2) if the item corresponded to the dimension in which it was included; (3) if the order in which it appeared in the questionnaire was considered correct; and (4) if the item was easy to understand. Items that were considered irrelevant or unimportant were eliminated and the wording of items that were difficult to understand were revised. A new version of 58 items was obtained after removing the following 21 items: two items from the “planning intervention” dimension, three items from the “professional communication” dimension, three items from the “clinical responsibility” dimension, 6 items from the “self-appraisal and professional development” dimension and seven items from the “evidence-based practice” dimension. In the fourth week of September 2019, a virtual meeting with the same professionals was conducted. After including three new items (one item in the “delivering intervention” dimension and two items in the “clinical responsibility” dimension) and removing 15 items (three items from the “observation and assessment” dimension, two items from the “planning intervention” dimension, one item from the “delivering intervention” dimension, two items from the “professional communication” dimension, three items from the “clinical responsibility” dimension and four items from the “self-appraisal” dimension), a final version of the questionnaire with 46 items was obtained. The questionnaire was named CPTO as the Spanish acronym for the “assessment of professional competencies in occupational therapy”. None of the participants in this study received any kind of compensation.

### 2.3. Participants

The sample consisted of 295 students who were evaluated by occupational therapists in a practical setting. In order to be part of this study, occupational therapists were required to have the following: (1) at least 2 years of clinical experience; (2) with a minimum of 200 h in contact with students in order to assess the development of their professional skills. The fieldwork was carried out between September 2018 and June 2021.

### 2.4. Data Collection

The questionnaire was completed based on the information obtained by the therapists on different performance tests, evaluation of a patient, planning the intervention, developing the intervention, and on the observation made by the clinical tutors after a minimum of 200 h of direct contact with the students. The study was approved by the Ethics Committee on Human Research of the University of Granada (code 458/CEIH /2017).

### 2.5. Statistical Analyses

All statistical analyses were performed using IBM SPSS Statistics for Windows (version 26.0, IBM Corp., Armonk, NY, USA). The statistical significance was set at *p* < 0.05 (bilateral). Participant characteristics were analyzed using simple descriptive statistics.

#### 2.5.1. Development of the Final Questionnaire and Internal Consistency

An exploratory factor analysis was performed to identify the domains, reduce the number of items if possible, and determine which of them should be retained [13,16]. To decide the relevance of factor analysis, we estimate Kaiser–Meyer–Olkin’s sample adequacy statistic (acceptable for values >0.5) and the Barlett sphericity test. The structure was evaluated by means of an exploratory factorial analysis by varimax rotation, with the extraction of maximum likelihood, and applying the rule of self-values >1.8 to determine the number of factors. Items were removed if they had factorial loads <0.4 with their own factor, or if they were not discriminatory for having similar factorial loads in several factors. The process for deleting items was to delete them one by one by performing a factorial analysis repeatedly at each step. The answer options for each item were based on a six-point ordinal scale (1 = never; 2 = rarely; 3 = sometimes; 4 = half the time; 5 = almost always; 6 = always). The higher score, the better level of competencies. To determine the internal consistency (that is, the homogeneity of items that measure the same attribute), Cronbach’s alpha was calculated for the questionnaire and for each of the factors found in the factor analysis. In general, a Cronbach alpha of 0.70–0.95 was considered to correspond to a good internal consistency.

#### 2.5.2. Floor and Ceiling Effects

In this study, the floor and ceiling effects refer to the percentage of students who scored as high or low as possible. The percentages of the study were calculated with the highest and lowest possible scores in the total CPTO and every dimension. These effects were considered present when 15% of participants had minimum or maximum scores, which reduces the reliability of the instrument, as participants with extreme scores cannot distinguish themselves from each other.

#### 2.5.3. Interpretability

The difference in the total CPTO score and score of each of its three factors was analyzed using Mann–Whitney U test. Following the methodology for calculating quartiles for grouped data [17,18], the quartiles were determined to know the distribution of the students in each of the factors of the CPTO questionnaire according to the score achieved, obtaining four categories of scoring (low, medium, high or exceptional).

## 3. Results

### 3.1. Participants

Two hundred and ninety-five students conducted the field work in the following several areas of the clinical setting: children (*n* = 74, 25.1%), physical dysfunction (*n* = 85, 28.8%); mental health and intellectual disability (*n* = 59; 20%), and the elderly (*n* = 77; 26.1%). All were in the fourth grade of occupational therapy and had not conducted fieldwork in any other course before.

### 3.2. Factor Analysis and Internal Consistency

Table 1 shows the results of the exploratory factor analysis, the factorial load of each of the items, the “missing” percentage, and Cronbach’s auto-values and alphas of the factors, as well as the variance explained after rotation. All elements of each factor showed a rotated factorial load greater than 0.4. All the factors had an own value greater than 20.46% and a Cronbach alpha of 0.80 or higher. The total percentage of variance explained after rotation was 70.22%. The final questionnaire, derived from the factor analysis, included 33 items, grouped into the following three factors: (1) self-appraisal and professional responsibility (12 items), explaining 24.35% of the variance; (2) communication skills and delivering intervention (11 items), which explains 23.16% of the variance; and (3) clinical reasoning for assessing and planning the intervention (10 items), which explained 22.70% of the variance. The reliability of the total scale was a Cronbach alpha of 0.949, and for the first factor, a Cronbach’s alpha was obtained at 0.951; for the second factor, the Cronbach’s alpha value was 0.944; and the last factor obtained a Cronbach alpha value of 0.947. In order to know if the factors maintained a theoretical relationship, a correlation was conducted with Spearman’s Rho test.

### 3.3. Discriminant Validity

Table 2 shows the Spearman’s correlations between the total score of CPTO and the score of each of its factors, with the scores of the clinical case and final mark in clinical practice. The results show that the total score obtained by CPTO was strongly and positively correlated to each of the scores. Likewise, all the factors correlated positively and significantly with the other factors. The three factors showed high correlation. Both the qualifications of the clinical case and the final mark of practices obtained by the student showed a greater correlation with the clinical reasoning factor.

### 3.4. Floor and Ceiling Effects

Table 3 shows the maximum and minimum scores of the CPTO questionnaire and its three factors, along with percentage of individuals with maximum and minimum scores. All the percentages were below 34%.

### 3.5. Interpretability

Table 4 shows the average scores obtained by excellent students, and by those with a median or low performance in global CPTO and in each of its three factors. As can be observed, there were significant differences between both groups. Cohen’s D values showed big differences in all the factors and in the total score of the questionnaire between the two groups.

**Figure 1:**
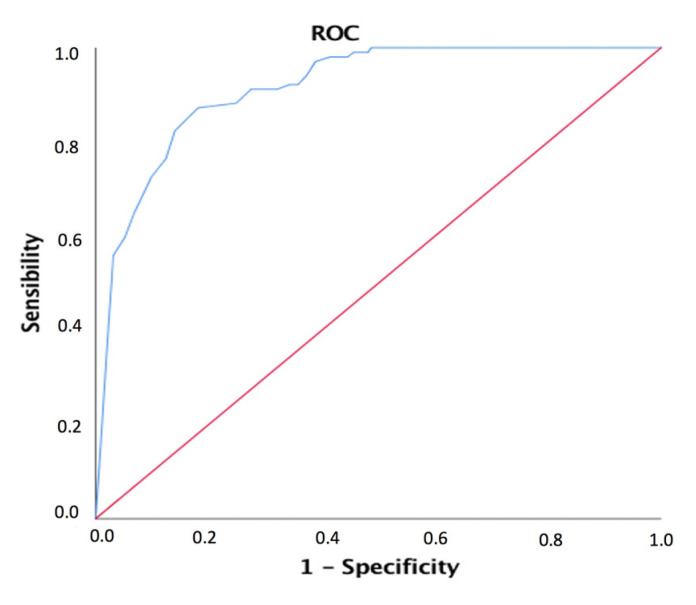
ROC curve used to determine the predictive value of the CPTO in determination of students with excellent skills.

Likewise, Figure 1 shows the ROC curve for the predictive level of CPTO in the determination of excellent skills. The area under the curve was 0.916 (95% CI: 0.884–0.947; *p* < 0.001). A score ≥152.50 on the CPTO indicates that the student has excellent skills.

Table 5 shows the scores for each quartile in each factor. From the quartiles, the variables were transformed after their visual grouping, obtaining the cut-off points for low, medium, high and excellent scoring.

## 4. Discussion

Professional practices are essential in the academic training of occupational therapists, constituting one of the most important subjects [19]. In this sense, an international study that was conducted, with students highlighting the central role that practical education and professional socialization plays in the formation of professional identity. The time spent on practical education in the ‘real world’ plays an implicit role in the formation of professional identity. In this study, we explore the psychometric properties of CPTO, a new tool for evaluating the professional skills of occupational therapy students during fieldwork. After analyzing their items, 33 of the original 79 items were retained in the final version. The results indicate that the questionnaire has good psychometric properties, in terms of validity, reliability, and discriminatory value for students with outstanding performance. In addition, the design allowed the development of cut scores for the CPTO.

### 4.1. Discriminatory Validity and Interpretability

The CPTO questionnaire and each of its factors showed good construct validity. This study provides preliminary evidence of the discriminatory validity of CPTO. The validity was demonstrated by the fact that this instrument shows differences between students with low, medium, high or excellent scores. The total score obtained for CPTO allows the students’ competencies to be consistently differentiated according to their score.

The dimensions of the CPTO questionnaire have common factors with other evaluation tools, such as professional behavior, self-management, communication, information gathering, and direct service provision, included in the SPEF-R2 [11]; clinical reasoning and communication are also included in CBFE and PEOTS [10]. All these factors that are collected are also perceived by occupational therapists as relevant to their practical training [19]. Occupational therapy professional behaviors include dependability, initiative, professional presentation, and organization [20], related to the third factor of CPTO; cooperation, empathy, verbal communication, and written communication are included in the second factor of CPTO; and the first factor includes the other two elements, as follow, described in occupational therapy’s professional conduct: the clinical reasoning and supervisory process.

According to the results of the distribution of scores in quartiles, it is observed that for the skills of self-appraisal and professional responsibility, more than half of the students achieve the maximum mark. It is worrying that around 26% scored low on this factor. These results are of interest because they suggest less autonomy when learning to learn, which is in accordance with the need to be aware of one’s own strengths and weaknesses. In addition, this factor reflects the dimension of professional responsibility, which may suggest the need to make students more aware of the responsibility and role they have in their professional practice.

Likewise, in the communication and delivering intervention skills, 25% of the students obtained a low score in these skills. Therefore, it would be interesting to consider whether, prior to the beginning of the internship period, practical training workshops on communication skills and therapeutic use of the self were implemented in future academic years and assess whether this dimension improves.

Clinical reasoning skills were the ones with the fewest number of students with the highest score. In this way, it can be understood that clinical reasoning is a more difficult skill to learn, for which students need more support. For this reason, we believe that it is convenient to start the study of clinical cases from the first formative stages and graduate this learning in a way that facilitates the acquisition of these skills. In this sense, the use of expert systems could be a good idea to complement learning, as happens in the training of other health professionals.

### 4.2. CPTO Questionnaire Description

The final questionnaire consisted of 33 items, which were grouped into three factors (Appendix A and Appendix B). The first factor explains the students’ performance in internships related to clinical responsibility and self-appraisal. Other authors have also indicated the importance of self-management, decision making, and professional behavior skills during their educational experience of professional practice [20], and their relationship to communication skills [21]. These types of competencies were also included within the professional competencies that regulate the profession of occupational therapists, such as professional autonomy [22], and in the model of professional behaviors [20]. In addition, it has been observed that practical training has a significant impact on the development of professional identity [23]. The development of a good professional identity is a protective factor against work stress and allows us to be resilient in the work context [24]. The second factor in this questionnaire refers to communication skills and delivering the intervention. This factor explains more than 23% of the variance regarding the student’s performance during their internship. Communication skills and their use in therapy are so relevant that specific models for occupational therapy have even been developed [25]. In fact, communication skills are also contemplated in other instruments, as in the SPEF-R2 [11]. Thus, communication skills can be understood as an essential element in the development of the professional competence of occupational therapists that has an impact both on relationships with other colleagues, on the establishment of a good therapeutic relationship, and on the greater satisfaction of patients and family members [21]. Our findings support the results of other studies that showed that interaction skills, such as active listening and empathy with the patient, facilitate intervention and patient-centered care [26]. In addition, communication skills have been linked to stress management skills, resilience, and emotional well-being for occupational therapy students [21]. Furthermore, the modes of communication in the field of occupational therapy can vary from culture to culture, as recent studies have shown [25]. That is why we think that having a culturally developed and validated instrument that includes this factor is a strong point of the CPTO instrument.

Regarding the third factor, clinical reasoning for assessing and planning interventions, it is considered one of the most important competencies for the professional performance of occupational therapists, who start to acquire it with the beginning of theoretical training and continue their learning during clinical practices. Clinical reasoning makes it possible to decide what and how to evaluate what is the best intervention, and, ultimately, formulate hypotheses that guide the therapeutic process [20,21,27]. Several internal and external factors have been described that influence the clinical reasoning process and should be taken into account in curricular practices, such as the following: (1) the emotional, cognitive and physical availability of the client (i.e., awareness of the difficulties, personality, educational level, learning style, and functional characteristics; (2) the prior knowledge that the client has and its physical characteristics; (3) the physical, cultural and social environment of the client; (4) the context in which the therapy is carried out (i.e., physical location, implicit rules, peers, etc.); (5) characteristics of the task (i.e., objective, degree of difficulty/security, etc.); (6) the interaction of factors (environment–client; task–environment); (7) knowledge and experiences of the therapist; (8) personal habits; and (9) preparation to develop therapy [6]. In this way, the CPTO allows these aspects in this factor to be considered, and it agrees with the previous literature [20] and legislation that includes the functions of occupational therapy [22,28]. Other authors have identified a number of determinants that positively influence the implementation of knowledge in occupational therapy practice, such as the following: (1) Adaptability, which is understood as the degree to which an intervention can be adapted. The more adaptable the environment, the more positive it is for learning; (2) the learning climate, if it promotes interprofessional collaboration, and the exchange of experiences and reflections in small groups in an environment without prejudice, or supported by a research team; (3) have the support of managers; (4) material and temporary resources available; (5) perception that the intervention is appropriate; (6) interest in continuous learning, to improve and develop new therapeutic strategies or guidelines; and finally, influences the (7) performance or execution of the therapy itself [29]. In this sense, the feedback and type of supervision carried out by the instructors play an essential role in learning [30] and in the development of the therapeutic style by the student, with the most frequent therapeutic modes being problem solving, collaborating, and instructing, and the less frequent ones being encouraging, empathizing, and advocating [31].

### 4.3. Implications for Practice

CPTO is a tool that could help the instructors become more aware of the importance of the different processes that influence the learning of professional skills and, in this sense, allow them to guide their students to improve their professional skills. Additionally, CPTO is an easy-to-complete tool that allows clinical supervisors to conduct an assessment easily.

### 4.4. Limitations and Future Work

This study has some limitations. The sample was obtained through a sampling of non-probabilistic convenience, among all clinical tutors and students of the University of Granada, which may imply a geographical bias, and this may affect the generalization of the results. Therefore, the study could be replicated in a larger sample, formed by students from different universities. Another limitation is that no test–retest evaluation was performed. Future scholarly studies may explore a test–retest evaluation. Regarding future lines of research, it would be interesting to perform studies in which a confirmatory factorial analysis of CPTO would be carried out in different areas of practice, such as the following: mental health, childhood, elderly, physical dysfunction, etc. Another possible limitation may be the time it takes to complete the questionnaire, which, for its final version, ranges from 30 to 45 min. Finally, the absence of related literature on the subject makes it difficult to discuss the results, since there are only a few tools designed to assess the clinical skills of occupational therapy students, so the impact of this type of tool on the student’s learning and evaluation process should be studied in depth.

## 5. Conclusions

The CPTO questionnaire makes a new contribution to the understanding of the development of the professional skills of occupational therapy students. CPTO is a tool that complements other tools that are used by clinical supervisors who are in charge of evaluating the progress of students during their practical training, and can be a useful instrument for teachers, since it facilitates knowing the progression and qualification in the fieldwork, allowing the detection of students with difficulties in their clinical performance, as well as those skills in which they need support to improve. It is also useful for students to know their own progress, and be more aware of their strengths and weaknesses, in order to achieve greater excellence and quality in care services. The psychometric results confirm the internal consistency of the instrument, as well as its construct validity and discriminatory validity, according to the information provided by the clinical tutors participating in the study. Finally, CPTO is the first questionnaire in Spanish, developed to assess professional skills. It is necessary to continue working on the formal incorporation of our CPTO questionnaire in the evaluation of students.

## Figures and Tables

**Table 1:** Exploratory factor analysis (33 items).

	1	2	3
**Factor 1. Self-Appraisal and Professional Responsibility (12 Items)**			
Demonstrates a good understanding of professional roles	0.791		
Shows initiative, disposition and reliability	0.736		
Uses resources rationally	0.703		
Meets the right professional responsibilities and is punctual	0.677		
Organizes and manages time effectively	0.646		
Manages the uncertainty inherent in clinical practice with prudence, knows his/her limitations and asks for help when the situation requires it	0.644		
Comments his/her own evolution with the clinical tutor.	0.629		
Identifies his/her own clinical strengths and needs for professional development	0.621		
Ensures patient safety	0.578		
Analyzes therapist–client interaction	0.576		
Makes decisions and selects a treatment plan agreed with the tutor	0.566		
He/she is aware of the impact of his/her own behavior on others	0.530		
**Factor 2. Communication Skills and Delivering Intervention (11 Items)**			
Builds a therapeutic relationship with the client/carers, professionals, etc.		0.779	
Communicates appropriately with the client/carers		0.770	
Works as a member of the team and establishes a good relationship with other colleagues		0.707	
Conducts the planned activities		0.700	
Selects the right materials		0.673	
Knows how to adapt to the client’s motivation		0.651	
Explains the intervention and justifies it to the client/carers		0.638	
Modifies goals as the client evolves		0.601	
Sets an appropriate pace in the intervention		0.572	
Provides feedback to the client, family or/and other professionals		0.502	
Modifies clinical practice and considers alternatives based on the tutor feedback		0.439	
**Factor 3. Clinical Reasoning for Assessing and Planning the Intervention (10 Items)**			
Performs a structured clinical observation of the client and relies on models			0.742
Hypothesizes the client’s strengths and weaknesses and selects appropriate assessment tools			0.738
Uses standardized and/or non-standardized evaluation tools			0.729
Adapts activities and/or occupations			0.666
Analyzes activities			0.661
Collects appropriate user’s information, consulting medical, professional, family histories, interviews, etc.			0.653
Reasons and concludes evaluation results			0.631
Identifies and establishes appropriate strategies for achieving the objectives			0.560
Sets the objectives (short and long term) and involves the client			0.560
Values the effectiveness of the intervention plan based on the client and/or family feedback			0.504
Extraction method: analysis of main components. Rotation method: Varimax with Kaiser standardization. Rotation has converged into 6 iterations.			

**Table 2:** Relations between CPTO and clinical case and final mark on practice.

	Factor 1	Factor 2	Factor 3	CPTO	Clinical Case	Mark on Practice
Factor1		0.857 **	0.858 **	0.938 **	0.726 **	0.777 **
Factor2			0.855 **	0.941 **	0.698 **	0.775 **
Factor3				0.957 **	0.770 **	0.814 **
CPTO					0.778 **	0.830 **
Clinical Case						0.811 **

** *p*-value < 0.01 (bilateral).

**Table 3:** Floor and ceiling effects: percentage of values in the minimum and maximum.

	Mean	SD	Min	Max	*n* in Min	*n* in Max	% in Min	% in Max
Score factor 1	53.65	8.96	14	60	1	100	0.3	33.9
Score factor 2	48.74	8.34	8	55	1	82	0.3	27.8
Score factor 3	43.21	8.01	14	50	1	85	0.3	28.8
Total Score	145.60	24.15	41	175	1	63	0.3	21.4

SD: standard deviation.

**Table 4:** Mean scores in students with and without excellent professional skills.

	Students with Low and Medium Performance in Professional Skills			Students with Excellent Performance in Professional Skills					
	*n*	Mean	SD	*n*	Mean	SD	Dif Mean	*d* Cohen	*p*
Factor 1	193	50.69	9.8	102	59.25	1.5	8.56	1.21	<0.001
Factor 2	193	45.97	9.1	102	53.98	1.8	8.011	1.22	<0.001
Factor 3	193	40.15	8.3	102	49.01	1.8	8.865	1.47	<0.001
CPTO	193	136.81	25.63	102	162.25	4.6	25.437	1.38	<0.001

**Table 5:** Quartiles scoring for each factor in the sample.

	Score	*n*	%
**Self-appraisal and professional responsibility**			
Quartile 1	≤51.00	78	26.4
Quartile 2	52.00–54.00	31	10.5
Quartile 3	55.00–57.00	35	11.9
Quartile 4	≥58.00	151	51.2
**Communication skills and delivering intervention**			
Quartile 1	≤45.00	75	25.4
Quartile 2	46.00–48.00	23	7.8
Quartile 3	49.00–52.00	41	13.9
Quartile 4	≥53.00	156	52.9
**Clinical reasoning for assessing and planning the intervention**			
Quartile 1	≤40.00	81	27.4
Quartile 2	41.00–43.00	35	11.9
Quartile 3	44.00–47.00	38	12.9
Quartile 4	≥48	141	47.8
**CPTO**			
Quartile 1	≤134,00	74	25.1
Quartile 2	135.00–144.00	32	10.9
Quartile 3	145.00–155.00	34	11.5
Quartile 4	≥156	155	52.5

## Data Availability

The data presented in this study are available on request from the corresponding author. The data are not publicly available due to privacy reasons.

## Appendix A. Assessment of Professional Skills of Occupational Therapy Students. Evaluación de las Competencias Profesionales en Estudiantes de Terapia Ocupacional (CPTO) (Spanish Version)

Por favor, indica con qué frecuencia el estudiante muestra las siguientes competencias profesionales, de acuerdo con los siguientes criterios:

Nunca = 1; Casi nunca = 2; A veces = 3; La mitad de las veces = 4; Casi siempre = 5; Siempre = 6

**Auto-valoración y responsabilidad profesional**

1. Demuestra un buen entendimiento de los roles profesionales.
2. Muestra iniciativa, disposición y fiabilidad.
3. Hace un uso racional de recursos.
4. Cumple con las responsabilidades profesionales indicadas y es puntual.
5. Se organiza y maneja el tiempo de forma efectiva.
6. Maneja con prudencia la incertidumbre inherente a la práctica clínica, conoce sus limitaciones y pide ayuda cuando la situación lo requiere.
7. Comenta/discute su propia evolución con el tutor de prácticas.
8. Identifica sus propias fortalezas clínicas y necesidades para el desarrollo profesional.
9. Garantiza la seguridad del paciente.
10. Analiza la interacción del terapeuta–usuario.
11. Toma decisiones y selecciona un plan de tratamiento consensuado con el tutor.
12. Es consciente del impacto de su propio comportamiento en los otros.

**Comunicación y desarrollo de la intervención**

13. Construye una relación terapéutica con el usuario/cuidadores, profesionales, etc.
14. Se comunica adecuadamente con los usuarios/cuidadores.
15. Trabaja en equipo y establece una buena relación con otros colegas.
16. Realiza las actividades planificadas.
17. Selecciona los materiales adecuados.
18. Sabe adaptarse a la motivación del paciente.
19. Explica la intervención y lo justifica a los usuarios/cuidadores.
20. Modifica los objetivos según evoluciona el usuario.
21. Establece un ritmo adecuado en la intervención.
22. Proporciona feedback al usuario, familia y/u otros profesionales.
23. Modifica la práctica clínica y considera alternativas en función del feedback del tutor.

**Razonamiento clínico para la evaluación y planificación de la intervención**

24. Realiza una observación clínica estructurada del usuario y se apoya en modelos teóricos para la práctica clínica.
25. Formula hipótesis sobre las fortalezas y debilidades del usuario y selecciona las he-rramientas de evaluación apropiadas.
26. Utiliza los instrumentos de evaluación estandarizados y/o no estandarizados adecuados.
27. Adapta las actividades y/u ocupaciones.
28. Analiza las actividades.
29. Recopila información adecuada del usuario, consultando historias clínicas, profesionales, familia, entrevistas, etc.
30. Razona y concluye resultados de la evaluación.
31. Identifica y establece las estrategias adecuadas para la consecución de los objetivos.
32. Plantea los objetivos (a corto y largo plazo) e involucra a los usuarios en los mismos.
33. Valora la eficacia del plan de intervención teniendo en cuenta el feedback del usuario y/o familia.

## Appendix B. Assessment of Professional Skills of Occupational Therapy Students (English Version)

Please, indicate how often the student shows the following professional competencies, according to the following criteria:

1 = never; 2 = rarely; 3 = sometimes; 4 = half the time; 5 = almost always; 6 = always.

**Self-appraisal and professional responsibility**

1. Demonstrates a good understanding of professional roles.
2. Shows initiative, disposition and reliability.
3. Uses resources rationally
4. Meets the right professional responsibilities and is punctual.
5. Organizes and manages time effectively.
6. Manages the uncertainty inherent in clinical practice with prudence, knows his/her limitations and asks for help when the situation requires it.
7. Comments his/her own evolution with the clinical tutor.
8. Identifies his /her own clinical strengths and needs for professional development.
9. Ensures patient safety.
10. Analyzes therapist-client interaction.
11. Makes decisions and selects a treatment plan agreed with the clinical tutor.
12. He/she is aware of the impact of his/her own behavior on others.

**Communication skills and delivering intervention**

13. Builds a therapeutic relationship with the client/ carers, professionals, etc.
14. Communicates appropriately with the client / carers.
15. Works as a member of the team and establishes a good relationship with other colleagues.
16. Conducts the planned activities.
17. Selects the right materials.
18. Knows how to adapt to the client’s motivation.
19. Explains the intervention and justifies it to the client /carers.
20. Modifies goals as the client evolves.
21. Sets an appropiate pace in the intervention.
22. Provides feedback to the client, family or / and other professionals.
23. Modifies clinical practice and considers alternatives based on the tutor feedback.

**Clinical reasoning for the evaluation and planning of the intervention**

24. Performs a structured clinical observation of the client and relies on models.
25. Hypothesizes the client’s strengths and weaknesses and selects appropriate assessment tools.
26. Uses standardized and/or non-standardized evaluation tools.
27. Adapts activities and/or occupations.
28. Analyzes activities.
29. Collects appropriate user’s information, consulting medical, professional, family histories, interviews, etc.
30. Reasons and concludes evaluation results.
31. Identifies and establishes appropriate strategies for achieving the objectives.
32. Sets the objectives (short and long term) and involves the client.
33. Values the effectiveness of the intervention plan based on the client and/or family feedback.
